# Elucidating how the red imported fire ant (*Solenopsis invicta*) diffused spatiotemporally among different landscapes in north Taiwan, 2008–2015

**DOI:** 10.1002/ece3.8465

**Published:** 2021-12-20

**Authors:** Chia‐Hsien Lin, Tzai‐Hung Wen, Yi‐Huei Liu, Rong‐Nan Huang, Helen Kang‐Huey Liu

**Affiliations:** ^1^ Department of Geography National Taiwan University Taipei Taiwan; ^2^ Department of Entomology National Taiwan University Taipei Taiwan; ^3^ Department of Political Science National Taiwan University Taipei Taiwan

**Keywords:** invasive species, land use, network analyses, red imported fire ant, spatial diffusion, Taiwan

## Abstract

*Solenopsis invicta* Buren, also known as the red imported fire ant (RIFA), has had a large negative impact on human and livestock health. However, few studies have further investigated the influence of human land use, which is an important factor affecting the habitats of insects, on the expansion of RIFAs. In addition, there is a lack of knowledge of the empirical associations between RIFA diffusion and land use within countries. Therefore, the objectives of this study were to provide an approach to delineate the areas of RIFA infestations and explore how land use influences the spatiotemporal diffusion of *S*. *invicta*. We used RIFA data from 2008 to 2015 from the RIFA surveillance system, which was conducted by the National RIFA Control Center in Taiwan. Two regions in Taiwan with different RIFA infestation levels were investigated. The ordinary kriging method was applied to show the spatial intensity of RIFAs, and the extreme distance estimator method was applied to determine the critical dispersal distances, which showed the distance of the highest probability of RIFAs in two consecutive years. In addition, network analyses were used to identify RIFA invasion routes between land‐use types. Finally, bivariate local indicators of spatial association were used to capture the invasion process in time and space. The results showed, paddy fields, main roads, and warehouses were identified as the top three land‐use types of diffusion sources. On average, the critical RIFA dispersal distances were 600 and 650 m in two consecutive years in high‐ and low‐infestation regions, respectively. Finally, RIFAs were likely to diffuse between main roads and warehouses in the low‐infestation region. Therefore, it is suggested that RIFA control activities be implemented at least 600 m from the observed spot. Additionally, control activities should be conducted on the identified three land‐use types of diffusion sources in the high‐infestation region, and the roadsides between main roads and warehouses in the low‐infestation region to prevent the accidental spread of RIFAs.

## Graphical abstract

Hot spot and invasive areas of RIFAs in Taoyuan by years: (a) 2012–2013, (b) 2013–2014, and (c) 2014–2015. Red blocks are hot spots, indicating high intensity of *S. invicta* for both consecutive years. Blue blocks represent diffusion areas that had a significantly higher intensity of *S. invicta* for the later year.

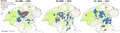

## 1 INTRODUCTION

*Solenopsis invicta* Buren, also known as the red imported fire ant (RIFA), has large negative impacts on biodiversity, human health, and economic costs. Regarding biodiversity, RIFAs can reduce the populations of native invertebrates (Lei et al., 2019) and vertebrates (Wojcik et al., 2001). In terms of human health, some humans die after being stung. Finally, estimations of economic losses, including medical and pesticide costs, in the southeastern United States were over half a billion per year (Williams & DeShazo, 2004). Therefore, effective control strategies are desired.

To control domestic RIFA expansion, one of the major approaches is to identify its local migration paths. One of the factors that could contribute to RIFA domestic dispersal is human activities, such as farming, moving soil, and cutting grass (King & Tschinkel, 2008; Williams & DeShazo, 2004). Mathematical modeling and computer simulations, such as cellular automata (Scanlan & Vanderwoude, 2006) and agent‐based modeling (Keith & Spring, 2013), have been used to investigate the dispersal of *S*. *invicta*. These studies could capture the dispersal distances of *S*. *invicta* and estimate the changes in the geographic extent of *S*. *invicta*. However, they could not clarify the association between land use by humans and the dispersal of *S*. *invicta*. The effect of land use by humans on RIFA expansions is an important factor affecting insect habitats (Carré et al., 2009; Tscharntke et al., 2008; Woltz et al., 2012).

In Taiwan, *S*. *invicta* was first reported in Xipu village, Taoyuan region, in October 2003 (Huang, 2004). To monitor RIFA occurrences and distributions, the Bureau of Animal and Plant Health Inspection and Quarantine (BAPHIQ), Council of Agriculture in Taiwan, launched a surveillance system in March 2004 (NRIFACC, 2020). To date, Taoyuan is the most infested region in Taiwan.

Control strategies in Taiwan are based on RIFA guidelines, which mainly focus on the use of different pesticides (NRIFACC, 2020). There are no evidence‐based suggestions regarding how large of an area or what land‐use types should be quarantined. Therefore, the objectives of this study were to provide an approach to delineate the areas of RIFA infestations and explore how land use spatiotemporally affects *S*. *invicta* diffusion. We examine the possible pathways of the spatiotemporal diffusion of RIFAs within regions and across land‐use types. This study provides approaches to clarify how human land use contributes to *S*. *invicta* dispersal and its dispersal pathway.

## 2 MATERIAL AND METHODS

### 2.1 Study area

This study involved two regions, Taoyuan and Hsinchu, which have different severities of RIFA invasion. Both regions belong to the subtropical monsoon climate. In Taoyuan, during the study period between 2008 and 2011, the highest annual temperatures were during June–September (monthly mean temperature range 25.9°C–29.9°C), and the lowest temperatures were recorded in January (mean temperature 14.9°C). In Hsinchu, during 2012–2015, the highest monthly mean temperatures ranged from 27.8°C to 30.4°C during June–August, while the lowest mean temperature (15.8°C) occurred during January–February (CWB, 2020). In general, the temperatures in these two areas are suitable for RIFA development (Cokendolpher & Francke, 1985).

The Taoyuan region, located in northwestern Taiwan (24°95′N, 121°23′E), has a population of 2.2 million and a population density of 1800.0 people/km^2^ in an area of 1180 km^2^. Taoyuan hosts the largest and busiest international airport in Taiwan (Taiwan Taoyuan International Airport), which is also one of the largest airports in the world. In addition, air cargos are imported globally. Thus, Taoyuan provides great opportunities for RIFA invasion from foreign countries. Since *S*. *invicta* was introduced in 2003, this species has gradually spread throughout the Taoyuan region. By 2008, Taoyuan had more than half of the RIFA‐infested area in Taiwan (>4000 ha; Kuo, 2008). Therefore, Taoyuan represents a region with a high level of RIFA infestation in this study.

**FIGURE 1:**
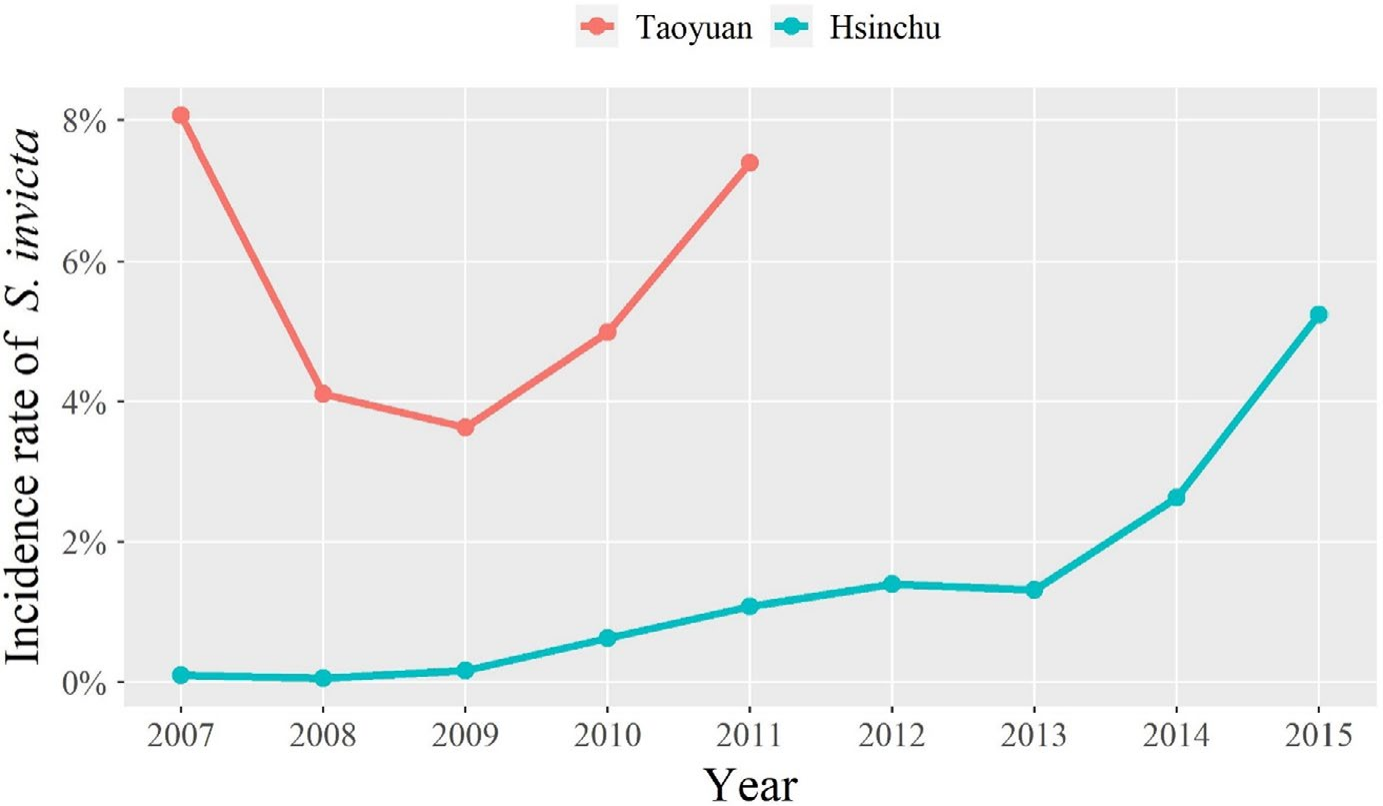
Incidence rate of *Solenopsis invicta* in Taoyuan (2007–2011) and Hsinchu (2007–2015). The incidence rate is defined as the percentage of positive tubes. Taoyuan is the region that was the first reported *S*. *invicta* infestation in Taiwan. The species that was introduced in Hsinchu, neighbors Taoyuan, has gradually spread over the past decade (2007–2015)

The Hsinchu region, including Hsinchu City and Hsinchu County, has a population of approximately 1.0 million and a population density of 66.0 people/km^2^. The region neighbors Taoyuan. In Hsinchu, RIFA invasions started in 2006 in the townships along the Hsinchu–Taoyuan border (Kuo et al., 2010), and this species has now been detected over the past decade (Figure 1). In this study, we explored RIFA data from 2012 to 2015 from the Hsinchu region. In comparison with Taoyuan, the RIFA situation in Hsinchu represented a low level of RIFA infestation region in this study (Figure 1).

### 2.2 Data

#### 2.2.1 *S*. *invicta* surveillance data

The RIFA data were obtained from the *S. invicta* surveillance system, which was conducted by the National RIFA Control Center in Taiwan. The system included coordinates of sample tubes, collection date, and presence or absence of *S*. *invicta* in tubes. The sample tube (12 cm long ×1.5 cm radius) was a RIFA trap. The tube contained a piece of potato chip on the top (Bao et al., 2011). Potato chips are a common method used for monitoring and detecting RIFAs (Bao et al., 2011; Lin et al., 2011; Stringer et al., 2011; Vogt et al., 2003; Yang et al., 2009), and they are a frequent method used in Taiwan for RIFA sampling (Lin et al., 2011; Yang et al., 2009).

**FIGURE 2:**
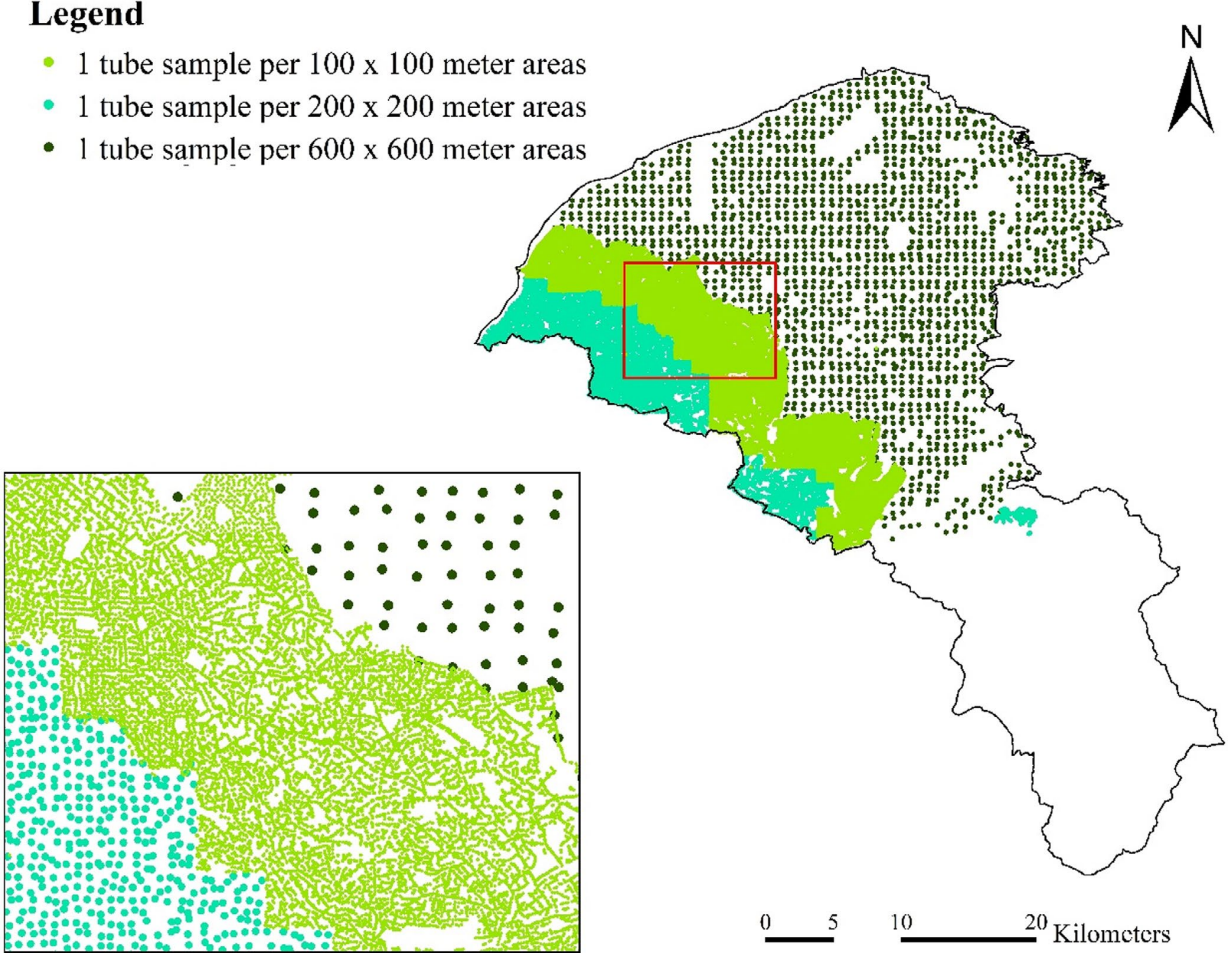
Tube samples with different sampling densities in Taoyuan, 2009–2011

The severity of the *S*. *invicta* invasion and the available budgets determined the sampling densities in the system. Sample tubes were systematically placed across the study areas except in mountain areas (Figure 2). In Taoyuan, one tube was placed per 200 × 200 m^2^ in 2008. In 2009, based on the 2008 RIFA data, the tube densities were adjusted to one tube per 100 × 100 m^2^ in highly invaded areas and one tube per 200 × 200 m^2^ was used in the remaining areas. In 2010, Taoyuan was further divided into high‐, middle‐, and low‐invasion areas (one tube per 100 × 100 m^2^, one tube per 200 × 200 m^2^, and one tube per 600 × 600 m^2^, respectively). The tube density was reset to one tube per 200 × 200 m^2^ in 2011.

Therefore, from 2008 to 2011, the overall number of tube samples in the *S*. *invicta* surveillance system in Taoyuan was 155,674. After removing inaccurate positions and missing information, 154,305 sample records were included in the study (Table 1). There were 8,003 tubes positive with positive RIFA occurrence (1861, 1941, 2785, and 1416 in 2008, 2009, 2010, and 2011, respectively).

**TABLE 1:** Summary of tube data in Taoyuan (2008–2011) and in Hsinchu (2012–2015)

Taoyuan						Hsinchu				
Year	2008	2009	2010	2011	Total	2012	2013	2014	2015	Total
+	1,861	1,941	2,785	1,416	8,003	224	178	377	742	1,521
‐	29,764	46,234	52,045	18,081	146,124	16,979	15,644	19,247	11,095	63,018
Total	31,625	48,175	54,830	19,497	154,305	17,203	15,822	19,624	11,835	64,539

Note

+ , positive tube; − , negative tube.

In Hsinchu, the sample density was uniform, with one tube per 200 × 200 m^2^ in each year during the study period of 2012–2015. There were 72,743 samples in total in the system, and 88.7% (64,539 samples) were valid samples that were included in this study (Table 1). There were a total of 1,521 tube samples with the presence of RIFAs (224, 178, 377, and 742 in 2012, 2013, 2014, and 2015, respectively).

#### 2.2.2 Land‐use data

The land‐use classification data conducted from 2011 to 2014 were used in this study (NLSC, 2015). These data have a three‐level classification system with a hierarchical tree structure. At level I, there are a total of nine land‐use types: agriculture, forest, transportation, water resources, buildings, public utility, recreation, salt mines, and others. At level II, there are a total of 41 more detailed land‐use types, which are derived from the level I land‐use types. At level III, there are a total of 103 land‐use types covering urban (e.g., warehouses, service industries, and residential areas) and rural (e.g., paddy fields, upland fields, and nurseries) land‐use types. Level III land‐use types were used in this study. After removing places that were not classified, such as mountain areas, the available land‐use data covered 47.3% of Taoyuan and 74.5% of Hsinchu.

### 2.3 Analytical approaches

A positive tube was defined as a tube that caught one or more *S*. *invicta* individuals. In this study, the number of positive tubes was analyzed.

However, the various tube sampling densities used during the study period may bias the RIFA results because there is a higher likelihood of tubes becoming positive in the areas with dense arrays than in the areas with sparse arrays. Therefore, the survey results could not be combined and compared. Thus, to analyze the tube data at various sampling densities, the tube data were standardized.

The tube data were standardized based on the likelihood that tubes became positive. A positive tube was set as one when the positive tube was within a density of 200 × 200 m^2^. When the positive tube was at a density of 100 × 100 m^2^, the tube was standardized as 0.25 by the formula (100/200)^2^. Furthermore, a positive tube was standardized to nine when it was within a density of 600 × 600 m^2^. The standardized number of positive tubes was used in the following analyses.

Our analytical framework included four major components. First, to illustrate the spatial intensity of *S*. *invicta*, the kriging method was applied. The kriging method is a spatial interpolation approach that uses point values to create a prediction surface in a study area (Sciarretta & Trematerra, 2014). Here, we applied the ordinary kriging method and used the standardized number of positive tubes as a point value for the given study year. The ordinary kriging method was applied using ArcGIS 10.7.

Second, to determine the geographic ranges of *S*. *invicta* diffusion, we calculated the distances between every two positive tubes between two consecutive years. The distances between every two positive tubes between two consecutive years mean that, for example, if we draw a circle with radius of 600 m from a positive tube in 2008, the circle will cover the sites with positive tubes in 2009. In this case, 600 m is the distance, and 2008 and 2009 are the two consecutive years.

The cumulative proportions of positive tubes between consecutive years at different distances were calculated. The extreme distance estimator method (Christopoulos, 2014) was used to determine the turning point of the cumulative proportion diagram as the critical dispersal distance of *S*. *invicta*. Here, the identified critical dispersal distance means the highest probability of the dispersal distance. Note that the identified critical dispersal distance did not equal to the largest dispersal distance. The largest dispersal distance means that the distance of the cumulative proportion of *S*. *invicta* occurrence between consecutive years reaches 100%. All calculations were performed in R version 3.6.2 by using the “rgeos” (Bivand & Rundel, 2020) and “inflection” (Christopoulos, 2019) packages.

Next, based on the previous results (i.e., critical dispersal distance), network analyses were used to identify RIFA invasion routes between land‐use types. In the network analyses, each land‐use type was represented by each node, and the links represented the diffusion relationship between land‐use types for two consecutive years. The sizes of the nodes were based on the hub values calculated by the hypertext‐induced topic selection (HITS) algorithm (Kleinberg, 1999). The land‐use types with the largest hub values indicated that *S*. *invicta* diffused from those areas to other land‐use types. In other words, the land‐use types with the largest hub values were viewed as diffusion sources. In addition, to identify the clusters of land use associated with the intensive diffusion of *S*. *invicta* among specific land‐use types, the network community detection method developed by Blondel et al. (2008) was used. The results meant that in the same cluster, *S*. *invicta* were likely to diffuse among the identified land‐use types; *S*. *invicta* were unlikely to diffuse among different clusters. All calculations were performed in R version 3.6.2 and Gephi version 0.9.2.

Finally, to capture the invasion process in time and space, the bivariate local indicator of spatial association (bivariate LISA) was used (Anselin et al., 2002). Based on the identified land‐use types with the largest hub values, we measured the spatial transition of *S*. *invicta* in the land‐use types for two consecutive years as follows:

$$I_{i,t}=\frac{\left(x_{i,t}‐\bar{x}\right)}{\delta_{x}}\sum_{j}w_{\mathrm{ij}}\frac{\left(y_{i,t+1}‐\bar{y}\right)}{\delta_{y}}$$

where *I_i_*_,_ *_t_* represents the bivariate LISA indicator at location *i* in year *t*, *x_i_*_,_ *_t_* represents the standardized number of positive tubes in the land‐use type with the largest hub values in year *t*, *y_j_*_,_ *_t_*_+_ *_1_* represents the standardized number of positive tubes in any land‐use type at location *j* in year *t* + 1, *δ_x_* and *δ_y_* are standard deviations, and *w_ij_* is the neighboring structure. Areas can be classified by the LISA indicator values into two distinct types of spatiotemporal significance: hot spots and diffusion areas. A positive *I_i_* value means that a certain location, *i*, and its neighboring areas represent a homogeneous cluster and have a higher tendency for local spatial dependency. Therefore, the areas with positive *I_i_* values are categorized as hot spot areas, capturing the sustained high intensity of *S*. *invicta* for consecutive years. In contrast, a negative *I_i_* value, which shows the opposite trend between *x_i_* and *y_j_* (i.e., a low *x_i_* value and a high *y_j_* value), implies negative spatial dependency (i.e., the area is a spatial outlier in relation to its neighbors). Thus, the areas with negative *I_i_* values were categorized as diffusion areas, indicating that its neighboring areas had a higher standardized number of positive tubes in the next year. A Monte Carlo significance test was used to evaluate the statistical significance of the spatial clusters and outliers. Bivariate LISA was calculated in GeoDa version 1.14.0 (Anselin et al., 2010).

## 3 RESULTS

### 3.1 Spatial intensity of *S. invicta*

**FIGURE 3:**
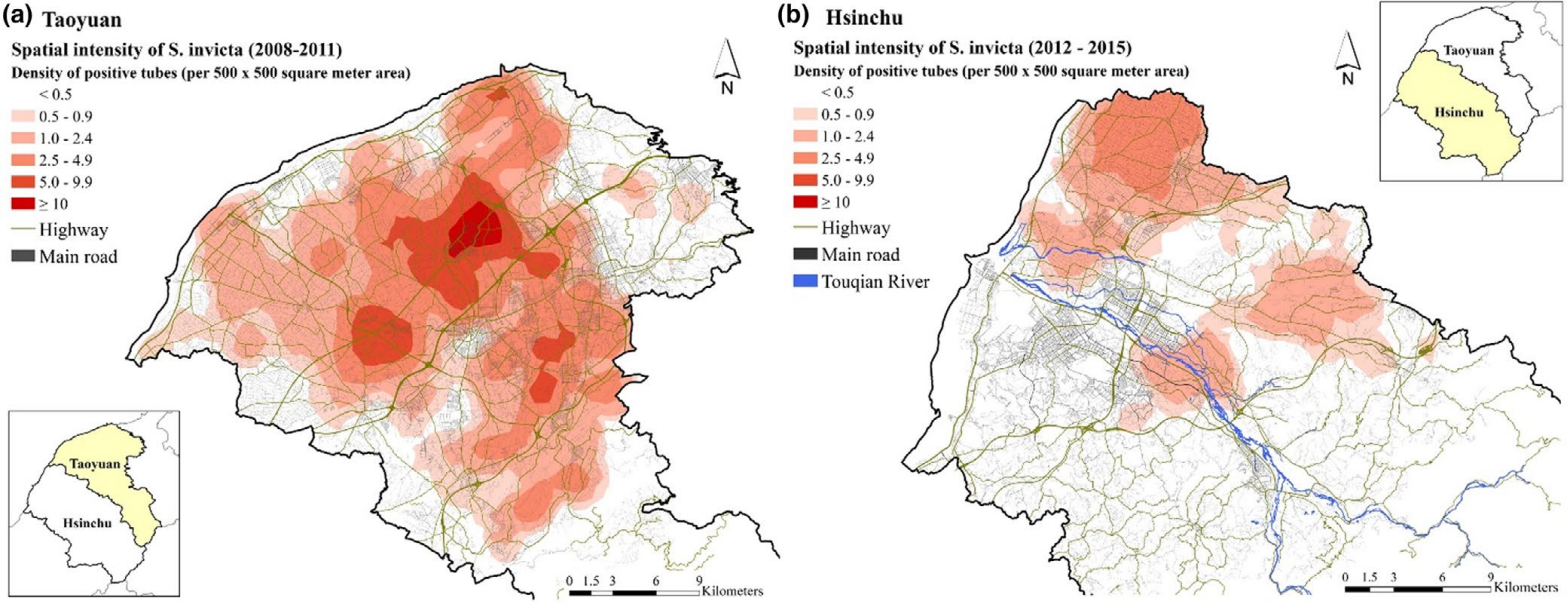
Spatial intensity of *Solenopsis invicta* occurrence in (a) Taoyuan and (b) Hsinchu. A positive tube was defined as a tube catching one or more *S*. *invicta*

The geographic areas affected by the spreading of *S*. *invicta* differed between Taoyuan and Hsinchu (Figure 3). It was estimated that *S*. *invicta* covered most areas of Taoyuan during 2008–2011 (Figure 3a). However, in Hsinchu, *S*. *invicta* occurred only in some areas connected with Taoyuan in the period from 2012 to 2015 (Figure 3b).

### 3.2 RIFA and land‐use types

**FIGURE 4:**
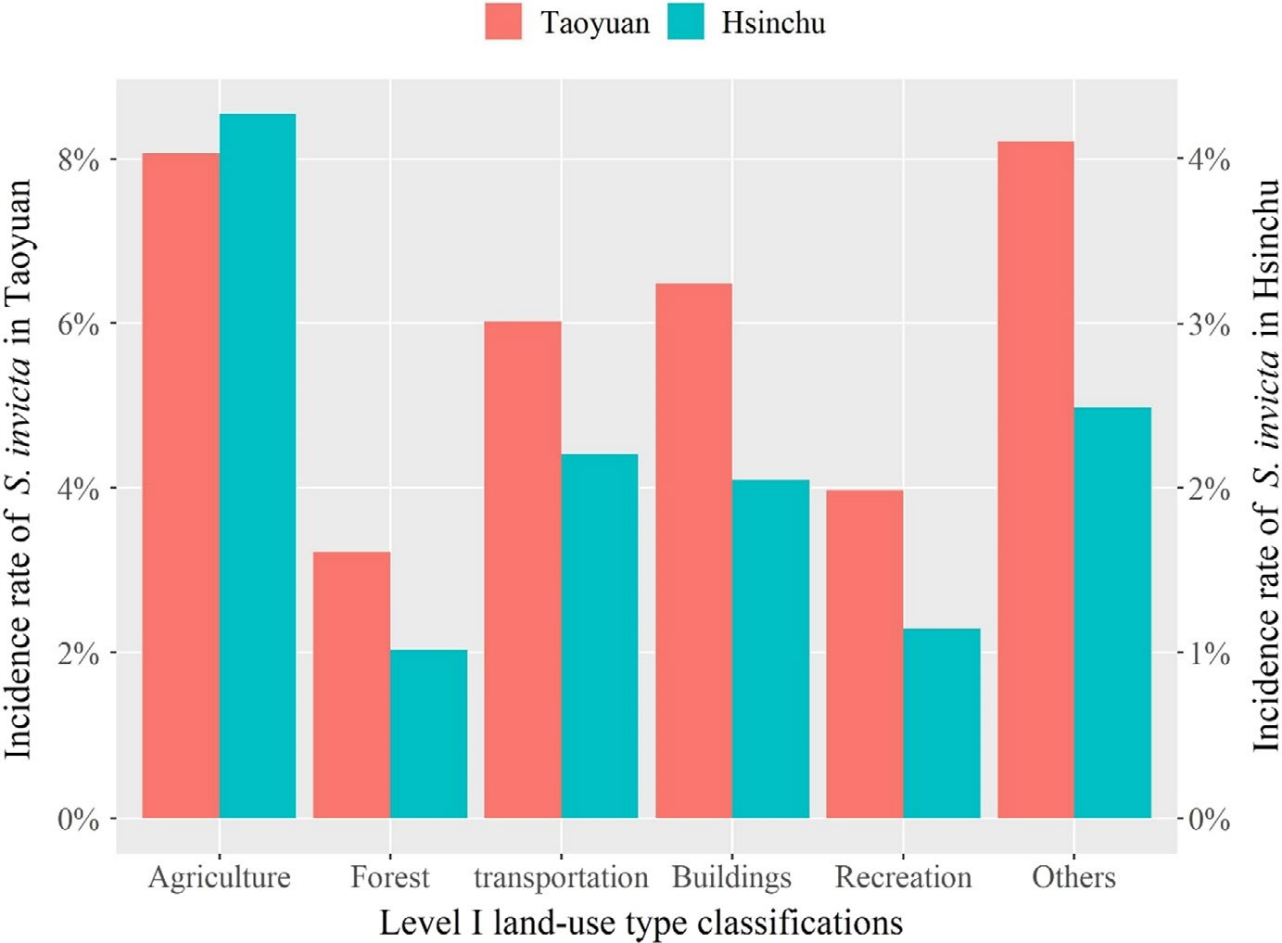
Average incidence rates of RIFA in different land‐use types in Taoyuan (2008–2011) and in Hsinchu (2012–2015). RIFA appears mostly on agriculture, transportation lands, and buildings. Incidence rate was calculated as the number of positive tube in the specific land‐use type divided by the number of overall (i.e., positive and negative) tube in the same land‐use type

Although there was a large difference in the standardized numbers of positive tubes between Taoyuan (*N* = 6812) and Hsinchu (*N* = 1521), agriculture, transportation lands, and buildings were the top three level I land‐use type classifications infested by RIFAs in both Taoyuan and Hsinchu (Figure 4). A similar pattern was found in the more detailed level III land‐use classification in Taoyuan and Hsinchu. *S*. *invicta was* mostly observed in paddy fields (49.7% and 62.4% of the agricultural land in Taoyuan and Hsinchu, respectively), on main roads (92.7% and 95.7% of the transportation land in Taoyuan and Hsinchu, respectively), and in warehouses (74.3% and 60.1% of the buildings in Taoyuan and Hsinchu, respectively).

### 3.3 Dispersal distances for the invasion of neighboring areas

**FIGURE 5:**
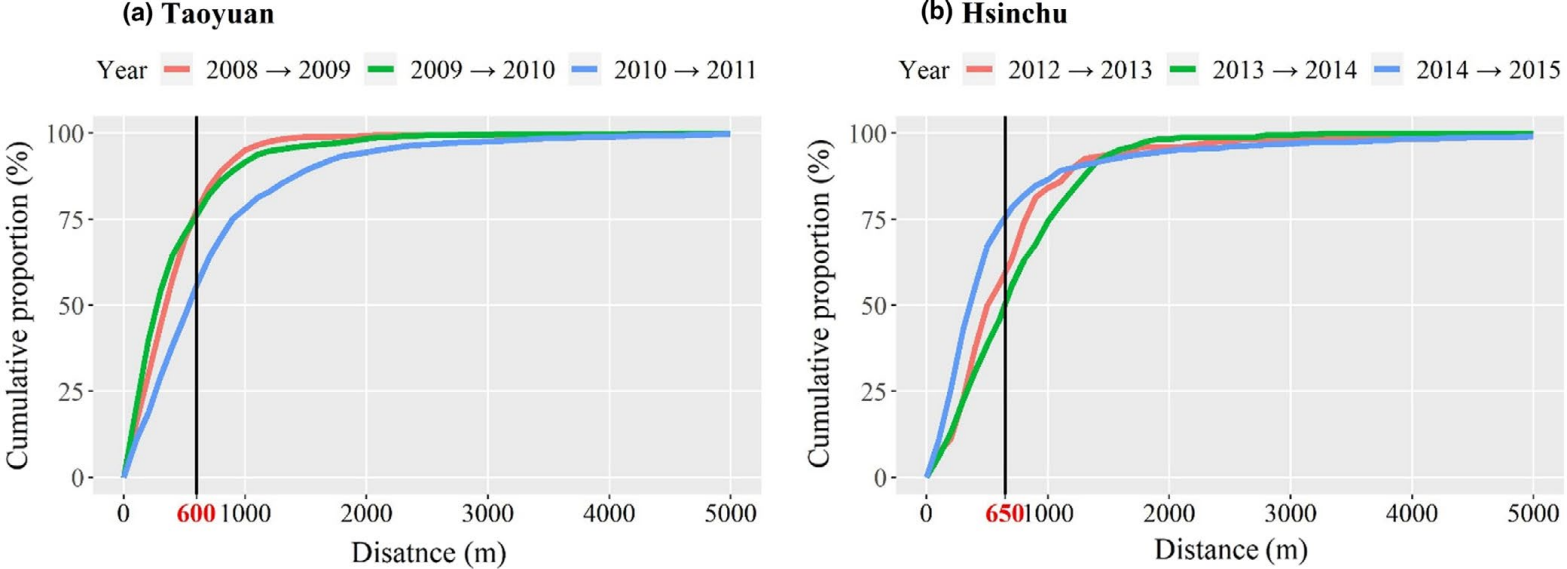
Cumulative proportion diagram of *Solenopsis invicta* occurrence under different dispersal distances in (a) Taoyuan and (b) Hsinchu. The dispersed distances of *S*. *invicta* were within approximately 1–5 km of the source nest in these two regions. The critical distances at the turning point are 600 and 650 m in Taoyuan and Hsinchu, respectively, which are regarded as the average dispersal distances of *S*. *invicta*

According to cumulative proportions, it showed the largest dispersal distance was from 1200 to 3200 m in Taoyuan (Figure 5a). In addition, the critical distance at the turning (i.e., critical dispersal distance) was 500 m from 2008 to 2009, 550 m from 2009 to 2010, and 750 m from 2010 to 2011 in Taoyuan (Figure 5a). Compared with Taoyuan, Hsinchu has a larger dispersal distance (i.e., from 2000 to 5000 m) (Figure 5b). The critical dispersal distance was 650 m from 2012 to 2013, 750 m from 2013 to 2014, and 550 m from 2014 to 2015 (Figure 5b).

Since the results in these two study regions showed similar critical dispersal distances in the four‐year periods, we adopted 600 and 650 m as the average critical dispersal distances of *S*. *invicta* occurrences for further analysis in Taoyuan and Hsinchu, respectively.

### 3.4 RIFA transition among different land‐use types

**FIGURE 6:**
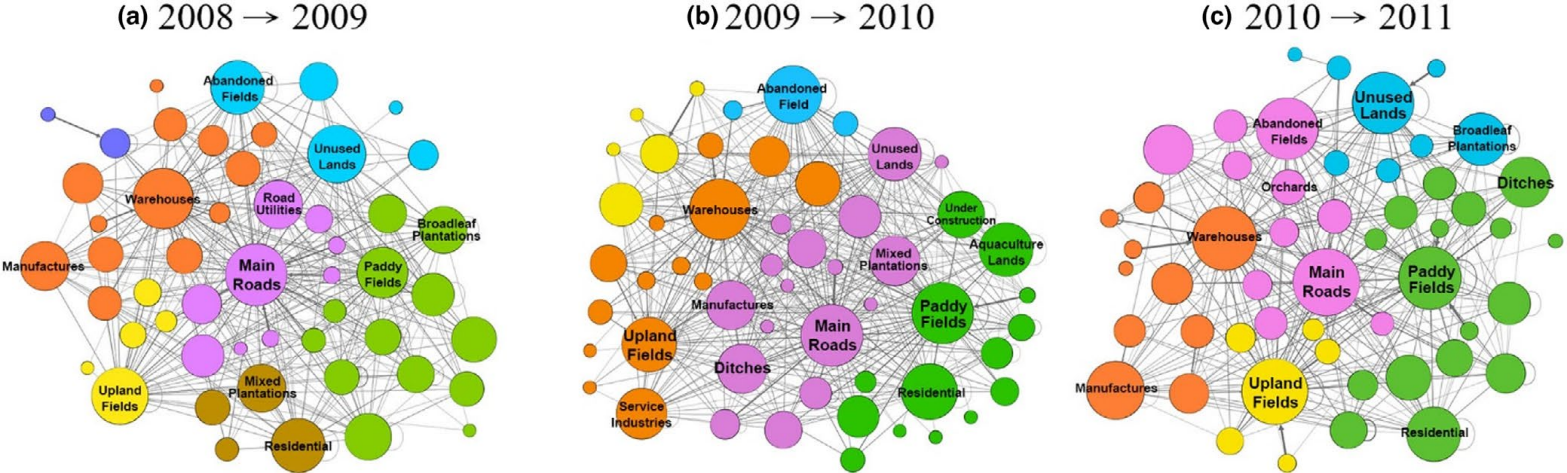
Diffusion relations among different land‐use types in Taoyuan by years: (a) 2008–2009, (b) 2009–2010, and (c) 2010–2011. The nodes represent all kinds of land use, and the links represent the diffusion relationship among land‐use types across different years, while thicker links mean higher transition frequencies between two land‐use types for consecutive years. The sizes of the nodes are based on the hub values calculated by the hypertext‐induced topic selection algorithm. Thus, a larger node indicates that a given land‐use type more easily diffuses *Solenopsis invicta* toward other land‐use types. The colors of the nodes illustrate groups based on modularity score. The nodes with matching colors reflect strong *S*. *invicta* diffusions among those nodes

We applied network analyses to depict the diffusion of RIFAs among different land‐use types. In Taoyuan, the main roads had the highest hub values during the two consecutive years (Table 2), which meant that RIFAs were likely to move from the main roads to other land‐use types. In addition, the hub values of paddy fields and warehouses were consistently high among the 102 land‐use types. Notably, the main roads, paddy fields, and warehouses belonged to different colors, showing that they were in different clusters from 2008 to 2009, 2009 to 2010, and 2010 to 2011 (Figure 6). This indicated that RIFAs were unlikely to diffuse among these three land‐use types.

**TABLE 2:** Hub values and ranks of paddy fields, main roads, and warehouses in Taoyuan (2008–2011) and in Hsinchu (2012–2015)

Hub value (Rank^a^)	Taoyuan			Hsinchu		
	2008–2009	2009–2010	2010–2011	2012–2013	2013–2014	2014–2015
Paddy fields	0.212 (7)	0.273 (2)	0.267 (4)	0.477 (1)	0.379 (1)	0.381 (2)
Main roads	0.274 (1)	0.277 (1)	0.292 (1)	0.446 (2)	0.349 (2)	0.423 (1)
Warehouses	0.266 (2)	0.268 (3)	0.276 (3)	0.000 (21)	0.252 (7)	0.123 (16)

^a^ The highest hub value between two consecutive years is ranked as 1. There are 103 land‐use types in total.

**FIGURE 7:**
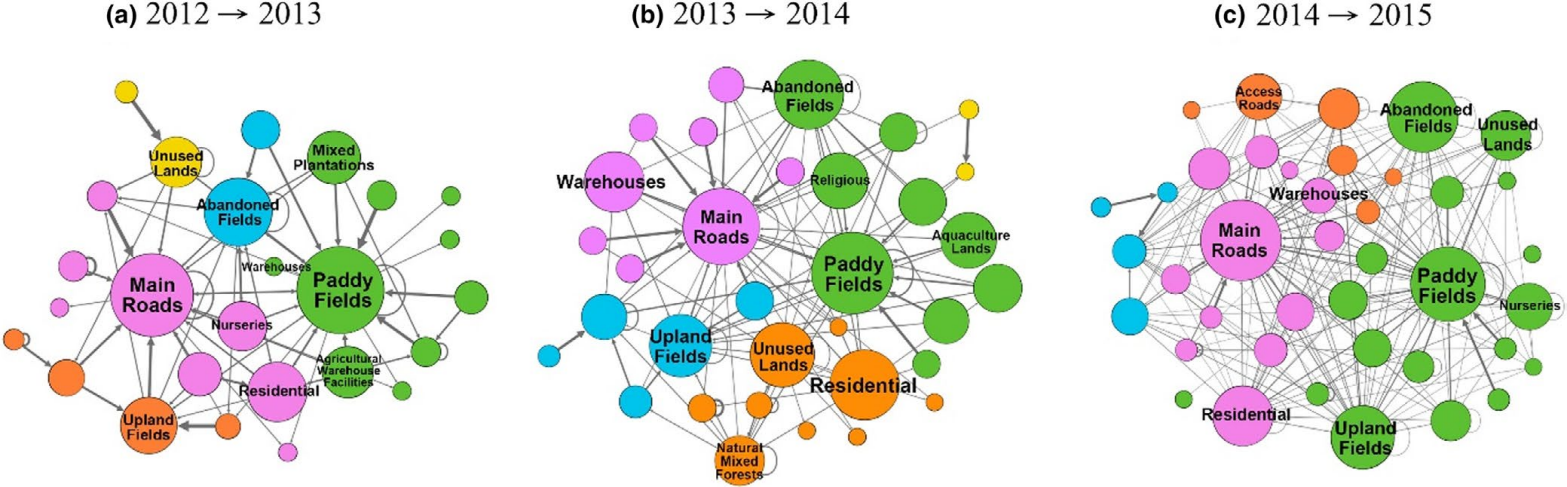
Diffusion relations among different land‐use types in Hsinchu by years: (a) 2012–2013, (b) 2013–2014, and (c) 2014–2015. The nodes represent all kinds of land use, and the links represent the diffusion relationship among land‐use types across different years, while thicker links mean higher transition frequencies between two land‐use types for consecutive years. The sizes of the nodes are based on the hub values calculated by the hypertext‐induced topic selection algorithm. Thus, a larger node indicates that a given land‐use type more easily diffuses *Solenopsis invicta* toward other land‐use types. The colors of the nodes illustrate groups based on modularity score. The nodes with matching colors reflect strong *S*. *invicta* diffusions among those nodes

In Hsinchu, paddy fields and main roads had the highest hub values during the study periods (Table 2), indicating that RIFAs tended to diffuse from paddy fields and main roads to other land‐use types. In terms of clusters, main roads and warehouses were clustered as a group from 2013 to 2014 (Figure 7b) and from 2014 to 2015 (Figure 7c).

### 3.5 RIFA invasion process through time and space

**FIGURE 8:**
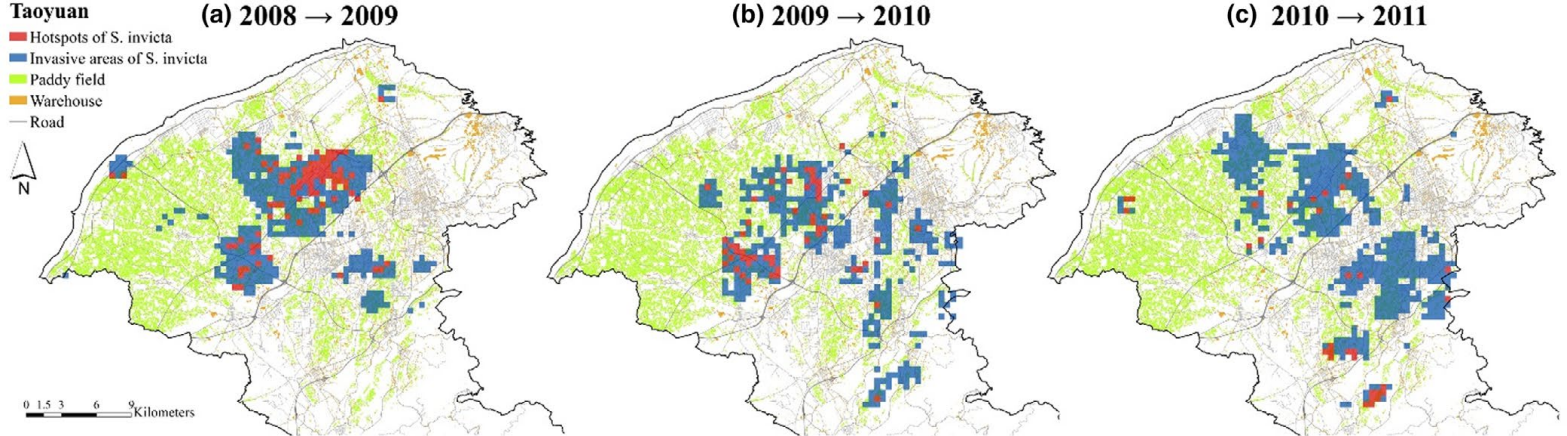
Hot spot and invasive areas in Taoyuan. Red blocks are hot spots, indicating high intensity of *S*. *invicta* for both consecutive years. Blue blocks represent diffusion areas that had a significantly higher intensity of *S*. *invicta* for the later year

**FIGURE 9:**
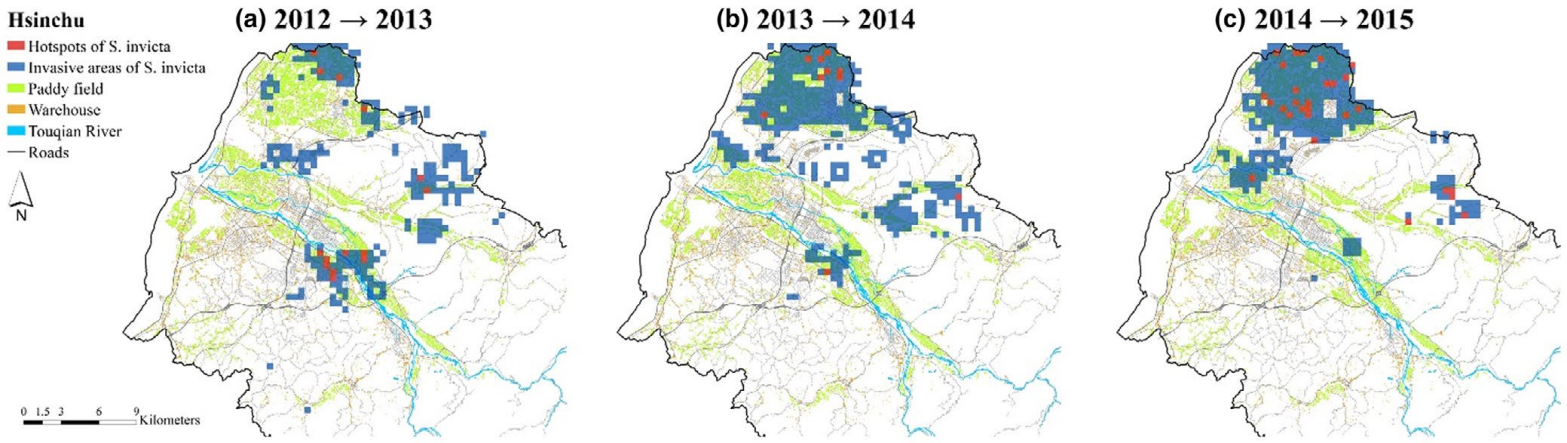
Hot spot and invasive areas in Hsinchu. Red blocks are hot spots, indicating high intensity of *S*. *invicta* for both consecutive years. Blue blocks represent diffusion areas that had a significantly higher intensity of *S*. *invicta* for the later year

As main roads had the highest hub values in most of the consecutive years in Taoyuan and Hsinchu (Table 2), we assumed that the main roads acted as the most important land‐use type of RIFA diffusion source. Therefore, we used the main roads and the surrounding areas to identify the hot spots and diffusion areas for consecutive years. The results showed that hot spot areas moved southward (Figure 8a, b) and that the diffusion areas were larger in Taoyuan (Figure 8c). In Hsinchu, at the beginning, hot spots were found in northern Hsinchu (Figure 9a), where the diffusion areas expanded (Figure 9b), whereas hot spots and diffusion areas in southern Hsinchu shrank (Figure 9c).

## 4 DISCUSSION

This study used comprehensive surveillance data to delineate the areas of RIFA infestations for profiling how land use spatiotemporally affects *S*. *invicta* diffusion. Our results indicated the longest dispersed distances of *S*. *invicta* were approximately 1–5 km of the source nest and the critical dispersal distances were 600 m in Taoyuan and 650 m in Hsinchu. Additionally, by formulating dispersal networks, this study identified main roads, paddy fields, and warehouses were three types of land‐use types as sources of *S*. *invicta* dispersal. In addition, *S*. *invicta* were likely to diffuse from main roads to warehouses in Hsinchu. Control strategies are suggested to be implemented based on the critical dispersal distances from source nests. Moreover, interventions were suggested to focus on main roads, paddy fields, and warehouses in Taoyuan, and the roadsides that were between main roads and warehouses in Hsinchu. Our findings provided a detailed understanding of the invasion process of RIFAs in different levels of RIFA infestations.

In this study, main roads were identified as an important land‐use type because they played a role as diffusion sources (Table 2). Our findings corresponded to a study conducted by King et al. (2009), who showed that roadway maintenance was a primary source of human‐mediated spread of polygyne fire ants. The main roads identified as diffusion sources could be because of high RIFA populations around main roads, and hence, RIFAs were likely to migrate from main roads to other land‐use types. High RIFA populations on main roads are shown in Figure 4, which shows that the RIFA incidence rates were high in the transportation category in both regions. It could be explained that roads are used to transport contaminated plants or soil. These contaminated soil may fall during transportation, further facilitating invasions near roadsides. Thus, high populations on the main roads fit RIFA ecology, as roads provide open and sunny environments for RIFA development (Gutrich et al., 2007; Wylie & Janssen‐May, 2017).

The results of network analyses showed that the main roads, paddy fields, and warehouses in Taoyuan belonged to different clusters, meaning that during the study period, *S*. *invicta* was unlikely to spread among these three land‐use types. One of the possible explanations was that the RIFA outbreak started in 2003 without effective controls; thus, *S*. *invicta* has become established in different niches in Taoyuan. Once *S*. *invicta* settles in an area, they disperse to the surrounding areas (Gutrich et al., 2007; Korzukhin et al., 2001). Another explanation was that the distance between paddy fields and warehouses in Taoyuan was over the critical dispersal distance (i.e., 600 m). As we applied the critical dispersal distance in network analyses to identify RIFA invasion routes between land‐use types, land‐use types beyond the critical dispersal distance were not included in the analysis.

The findings showed larger distances in the largest dispersal distance and critical dispersal distance in Hsinchu (Figure 5b) than in Taoyuan (Figure 5a). This could be due to the different combinations of social forms, monogynous and polygynous populations, in this two study regions. Although two social forms were not distinguished in this study, it could be expected that most colonies were polygynous colonies in Taoyuan due to a long and severe *S*. *invicta* infestation period (Chen et al., 2006). In addition, the finding from Yang et al. (2008) showed that central townships had more polygynous colonies than monogynous colonies in Taoyuan, but the patterns were opposite in the surrounding townships. Hsinchu is a neighboring region of Taoyuan, so it could be deducted that there are more monogynous colonies in Hsinchu, just like the surrounding townships of Taoyuan.

Paddy fields, main roads, and warehouses play different roles in Taoyuan and Hsinchu. In Taoyuan, all three land‐use types served as diffusion sources (Table 2). For example, main roads were likely to diffuse *S*. *invicta* to land‐use types such as unused lands and abandoned fields via roadsides as those were identified as a cluster (Figure 6). Note that *S*. *invicta* were not likely to diffuse among paddy fields, main roads, and warehouses. On the contrary, in Hsinchu, only main roads and paddy fields were identified as sources (Table 2). The difference in source lands could be due to different levels of RIFA infestations in these two regions (Figure 1). Therefore, by reducing RIFA incidence rates, it could prevent warehouses from becoming sources in Hsinchu. Findings from this study suggested that intervention focuses could be on the roadsides connecting between main roads and warehouses in Hsinchu, as main roads and warehouses were in the same cluster (Figure 7b and c).

The main roads and warehouses belonged to the same cluster in Hsinchu during the 2013–2014 and 2014–2015 periods (Figure 7). As the Hsinchu infestation was less severe than that in Taoyuan, *S*. *invicta* populations were not stable and were still in the process of growing and spreading from the main roads to warehouses. The findings from Hsinchu supported the idea that control measures should be comprehensively implemented in the early stage of invasion; otherwise, it will become difficult to eradicate species after they have spread and settled in an area (Hoffmann et al., 2011; Lodge et al., 2006; Simberloff, 2003).

Regarding the dispersal patterns in Hsinchu across three years, it was surprising to see a reduction in the southern part of Hsinchu (Figure 9). According to the BAPHIQ official reports in 2012, the authorities changed the control strategy used from large‐scale prevention to differentiated prevention (BAPHIQ, 2017). The differentiated strategy aims to contain the populations and development of *S*. *invicta* within the quarantine zone and allocate additional resources on sporadic occurrences outside the quarantine boundary to prevent their further spread southward. The areas of *S*. *invicta* reduction from 2012 to 2015 resulted from these policy changes and eradication efforts. Our results provide evidence that the differentiated strategy and precise treatment increased the success of *S*. *invicta* eradication in emerging areas.

For effective *S*. *invicta* control, it is important to establish appropriate treatment and monitoring zones. Previous literature shows that the natural dispersal distance of *S*. *invicta* is up to 5 km (Vogt et al., 2000), and monitoring should be conducted in a radius of 4 km for three consecutive years (Ujiyama & Tsuji, 2018). Our analysis of the geographic ranges of RIFA diffusion to neighboring areas across years confirmed that more than 99% of the nests found in a consecutive year had dispersed within approximately 2 km of the source nest, as shown in Figure 5. Additionally, the analyses showed that the most cost‐effective mean monitoring distance was 650 m in Hsinchu and 600 m in Taoyuan across years; these distances could cover nearly 75% of the range of *S*. *invicta* occurrences in years subsequent to initial invasions, as shown in Figure 5. Our findings help to quantify the specific distances that can be used as thresholds for implementing effective RIFA monitoring zones and control strategies in neighboring areas, especially when RIFAs start to invade new areas with diverse landscapes found in highly compact Asian regions.

There are some limitations of this study. First, we did not incorporate the effect of practical control measures in our analyses. The dispersal distance of *S*. *invicta* could more reasonably reflect natural invasions if adjusted for control measures. Second, we regarded the occurrence of *S*. *invicta* in a tube only as a positive record. In other words, the number of *S*. *invicta* in a tube was not counted in our study. Further investigation of the abundance of *S*. *invicta* over time and space is warranted. The two social forms of *S*. *invicta* were not identified in our samples. These distinct social forms may provide explanations for the spatial dispersal patterns of *S*. *invicta* in Taoyuan and Hsinchu. Moreover, Taiwan has annual heavy rains and typhoons. Dispersal distances of *S*. *invicta* could be increased by flooding. In addition, typhoons could destroy other ants’ habitats to facilitate *S*. *invicta* nesting. However, both factors were not considered in our study. Finally, in this research, an observational study was implemented to quantify the dispersal range of *S*. *invicta* to develop control measures. However, a randomized control study should be conducted to evaluate the effectiveness of control strategies for neighboring areas at various distances.

## CONFLICT OF INTEREST

The authors declare no conflict of interest.

## AUTHOR CONTRIBUTIONS

**Chia‐Hsien Lin:** Conceptualization (equal); Formal analysis (equal); Writing – original draft (equal); Writing – review & editing (equal). **Tzai‐Hung Wen:** Conceptualization (lead); Formal analysis (lead); Funding acquisition equal); Methodology (lead); Supervision (lead); Writing – original draft (lead); Writing – review & editing (lead). **Yi‐Huei Liu:** Investigation (equal); Methodology (equal). **Rong‐Nan Huang:** Data curation (lead); Supervision (supporting); Writing – review & editing (supporting). **Helen Kang‐Huey Liu:** Funding acquisition (lead); Resources (equal); Writing – review & editing (supporting).

## Data Availability

The authors archive the research data in the Dryad, a publicly accessible repository. The data can be accessed from https://doi.org/10.5061/dryad.vmcvdnctz.
